# Tackling the Taxonomic Challenges in the Family Scoliidae (Insecta, Hymenoptera) Using an Integrative Approach: A Case Study from Southern China

**DOI:** 10.3390/insects12100892

**Published:** 2021-10-01

**Authors:** Zhen Liu, Sheng-Jie Yang, Yu-Yuan Wang, Yan-Qiong Peng, Hua-Yan Chen, Shi-Xiao Luo

**Affiliations:** 1 College of Life and Environmental Sciences, Hunan University of Arts and Science, Changde 415000, China; qingniao8.27@163.com; 2 Key Laboratory of Plant Resources Conservation and Sustainable Utilization, South China Botanical Garden, Chinese Academy of Sciences, Guangzhou 510650, China; yangsj@scbg.ac.cn (S.-J.Y.); wangyuyuan95@scbg.ac.cn (Y.-Y.W.); luoshixiao@scbg.ac.cn (S.-X.L.); 3 CAS Key Laboratory of Tropical Forest Ecology, Xishuangbanna Tropical Botanical Garden, Chinese Academy of Sciences, Mengla 666303, China; pengyq@xtbg.ac.cn; 4 State Key Laboratory of Biocontrol, School of Life Sciences/School of Ecology, Sun Yat-sen University, Guangzhou 510275, China

**Keywords:** biological control, DNA barcoding, genitalia, morphology, pollinator, species delimitation

## Abstract

**Simple Summary:**

The scoliid wasps are larval parasitoids of scarabaeoid beetles and pollinators of various plants and therefore are important in biological control and pollination. However, some species of these wasps are extremely morphologically similar and difficult to identify. In this study, we used an integrative approach of species delimitation, combining morphology with molecular data, to investigate the species of Scoliidae from southern China. On the basis of both morphological and molecular evidence, we recognized 22 morphospecies of 9 genera in two tribes, among which one undescribed cryptic species of the polytypic species *Solia* (*Discolia*) *superciliaris* Saussure, 1864, five newly recorded species and one pending subspecies were discovered. Our study indicates that such an integrative approach is a potent tool in the effort to tackle the taxonomic challenges in Scoliidae, and even in other diverse groups of Aculeata, of which sexual dimorphism and cryptic species are common.

**Abstract:**

Species of the family Scoliidae are larval parasitoids of scarabaeoid beetles and pollinators of various plants. Despite their great importance in pest biological control and plant pollination, the taxonomy and systematics of these parasitoids are far from clear. Some species of the family are extremely morphologically similar and difficult to identify, especially in males. In this study, an integrative taxonomic approach, combining morphology and molecular data, was used to discriminate the species of Scoliidae from southern China. In total, 52 *COI* sequences belonging to 22 morphospecies of 9 genera in two tribes were obtained. The *COI* sequences worked well for the identification of all the studied species, with intraspecific genetic distances generally less than 2%, while interspecific distances ranged between 5.3% and 20.8%. The delimitations of the problematic species and subspecies of *Scolia* and *Megacampsomeris* are well solved by *COI* sequences, suggesting that DNA barcoding could be a useful identification tool for Scoliidae. Based on both morphological and molecular evidence, we discovered one undescribed cryptic species of the polytypic species *Solia* (*Discolia*) *superciliaris* Saussure, 1864, five newly recorded species, i.e., *Scolia* (*Discolia*) *sikkimensis* Bingham, 1896, *Sericocampsomeris flavomaculata* Gupta and Jonathan, 1989, *Megacampsomeris asiatica* (Saussure, 1858), *Megacampsomeris pulchrivestita* (Cameron, 1902) and *Megacampsomeris shillongensis* (Betrem, 1928) and one pending subspecies of *Scolia (Discolia) watanabei* (Matsumura, 1912) from China. Our study indicates that such an integrative approach, combing both molecular and morphological evidence, is a potent tool to tackle the taxonomic challenges in the family Scoliidae, or even, in other diverse groups of Aculeata, of which sexual dimorphism and cryptic species are common.

## 1. Introduction

Scoliid wasps (Hymenoptera, Scoliidae), commonly known as digger wasps or hairy wasps, form a medium-sized family with approximately 560 species worldwide. These wasps attack the mature larvae of scarabaeoid beetles, which are pests of agriculture and forestry, and some species have been considered as important biological control agents [1,2,3,4,5]. Some species have been imported and released in some countries, such as Australia, the Philippines and the United States, for the control of scarabaeoid larvae [6,7], at least one species—*Micromeriella marginella modesta* (Smith, 1855)—has been introduced and successfully established in Florida in the United States [7]. Females search for the location of scarabaeoid larva by vibratory signals, then they sting, paralyze and lay a single egg on the surface of their prey. The hatched scoliid larva develops externally and feeds on the tissues of the scarabaeoid grub mostly in the soil, though some develop in decaying wood or in rotten vegetation [8,9]. Laboratory observations have indicated that the life cycle of *Radumeris tasmaniensis* (Saussure, 1854) and *Radumeris radula* (Fabricius, 1775) lasts from 40 to 60 days, depending on season and species [2]. Scoliid adults of both sexes commonly visit the flowers of various plants, and some are found to be important pollinators [10,11,12,13,14]. Males of some scoliid wasps have been recorded as pseudocopulating with orchids. At least one species—*Pygodasis bistrimaculata* (Lepeletier)—may be the only pollinator of the terrestrial orchid *Geoblasta penicillata* (Chloraeinae) via pseudocopulation from subtropical South America [15]. *Scolia nobilitata* Fabricius, 1805 is the most common flower visitors in the buckwheat fields in north-central Florida [14]. Studies from Japan have suggested that scoliid wasps may play an important role in pollinating coastal plants in the grassland zone [11,12,13].

Despite their great importance, in the past few decades there have been few studies on this group from China since the rather comprehensive revisions of the Chinese Scoliidae made by Betrem [16,17]. Recently, Liu et al. [18] listed 1 subfamily (Scoliinae), 2 tribes (Campsomerini and Scoliini), 11 genera and 52 extant species in China, which provided a clearer view of the Chinese fauna. However, due to so many (overlapping) subspecies and varieties, many scoliid subspecies have been recognized merely on the basis of subtle color variations. In some cases, these subspecies are not represented by true geographically distinct populations and the boundaries between species or subspecies are often unclear. Furthermore, sexual dimorphism in Scoliidae is extreme; females and males of a single species often differ significantly, especially for species in the tribe Campsomerini. Males of the same species may vary significantly in size and color pattern and can be confused with closely related species. Further development of these important parasitoids as biological control agents against scarabaeoid pests and as pollinators in crop cultivation requires an accurate and sufficient identification of the species.

DNA barcoding (the partial sequencing of the gene cytochrome c oxidase 1, *COI*) has become an important species identification tool for insects [19,20]. The importance of the DNA sequences in the taxonomy and evolutionary relationships of Scoliidae or stinging wasps have been recognized by some researchers [21,22]. However, there is a serious lack of a DNA barcode database for the Chinese fauna of Scoliidae. To fill this gap, we started a research campaign with the objective of collecting specimens of Scoliidae across China in order to identify the species by morphological characters and to build a DNA database for these important parasitoids. By doing this, we aimed to test the morphological species delimitation of scoliid wasps from southern China using DNA barcoding methods.

## 2. Material and Methods

### 2.1. Collection and Identification

This work is based on specimens of Scoliidae collected by sweeping net and mostly Malaise traps (MT) set up in a few provinces of southern China. Specimens were identified using the keys of Liu et al. [23] All the studied specimens are deposited in the Museum of Biology at Sun Yat-sen University, Guangzhou, China (SYSBM). Multifocal images were made using a Nikon SMZ25 microscope with a Nikon DS-Ri 2 digital camera system. Images were then post-processed with Adobe Photoshop CS6 Extended.

### 2.2. DNA Extraction, Amplification, and Sequencing

In total, 52 specimens of 22 morphospecies were used for DNA barcoding analysis (Table 1, detailed collecting data of the sequenced see Table S1). Both female and male specimens were selected for a species when such specimens were available. Genomic DNA were extracted from the right mid leg of each specimen using the DNeasy Blood amd Tissue Kit (Qiagen, Hilden, Germany), following the manufacturer’s protocols. Following the extraction, the “barcode” region of the mitochondrial cytochrome oxidase subunit 1 (*COI*) was amplified using the LCO1490/HCO2198 primer pair [24]. Polymerase chain reactions (PCRs) were performed using Tks Gflex™ DNA Polymerase (Takara, Shiga, Japan) and conducted in a T100™ Thermal Cycler (Bio-Rad, CA, USA). Thermocycling conditions were: an initial denaturing step at 94 °C for 5 min, followed by 35 cycles of 94 °C for 30s, 50 °C for 30 s, 72 °C for 30s and an additional extension at 72 °C for 5 min. Amplicons were directly sequenced in both directions with forward and reverse primers on an Applied Biosystems (ABI) 3730XL by Guangzhou Tianyi Huiyuan Gene Technology Co., Ltd. (Guangzhou, China). Chromatograms were assembled with Geneious 11.0.3. All sequences generated from this study are deposited in GenBank (accession numbers see Table 1). All residual DNAs are archived (−30 °C) in the molecular laboratory of SYSBM, Guangzhou, China, and are available for further study upon request.

### 2.3. Sequence Analysis and Molecular Species Delimitation

All sequences were blasted in the BOLD (Barcode of Life Database, http://www.barcodinglife.org/index.php/IDS_OpenIdEngine) and GenBank (Accessed on: 9 August 2021). Sequences were aligned using MAFFT v7.470 by the G-INS-I strategy [25]. Genetic Kimura-2 parameter (K2P) distances within and between species were calculated in MEGA 7 with pairwise deletion for gaps [26].

Two methods, the Automatic Barcode Gap Discovery (ABGD) and the updated Poisson tree processes model (bPTP), were tested for molecular species delimitation. ABGD is a distance-based method and it sorts the sequences into hypothetical species by partitioning and comparing the difference between sequences to identify a “barcode gap” [27]. The ABGD analysis was performed on the web interface (https://bioinfo.mnhn.fr/abi/public/abgd/abgdweb.html. Accessed on: 10 August 2021), using the default priors, Pmin = 0.001, Pmax = 0.1, Steps 10, and with barcode relative gap width = 1.00. bPTP is an updated version of the original PTP with Bayesian posterior probability, which tests species boundaries on non-ultrametric phylogenetic trees by detecting significant differences in the number of substitutions between species and within species [28]. For bPTP analysis, a Maximum Likelihood (ML) tree was generated in RAxML v8.2.10 under the GTRGAMMA evolutionary model and performed on the bPTP web server (https://species.h-its.org/ptp/. Accessed on: 10 August 2021), with default parameters. The *COI* sequences of *Tiphia minuta* Linden (Hymenoptera: Tiphiidae; GenBank: JN299217) and *Vespa velutina* Lepeletier (Hymenoptera: Vespidae; GenBank: JKY224073) were selected as the outgroups because of their close relationships to Scoliidae [29].

## 3. Results and Discussion

The present study generated 52 *COI* sequences with an average of 678 bp. Voucher specimens of these 52 sequences were subjected to further morphological examination, and 22 species belonging to 9 genera were recognized (Table 1). When blasted in BOLD and GenBank databases, the sequences of the two *Scolia watanabei* specimens received close matches with *Scolia oculata* (Matsumura) (over 97%) and *Scolia hirta* (Schrank) (over 96%). Other species had no close matching sequences in BOLD or NCBI.

The K2P distances (Tables S2 and S3) showed a larger intergroup than intragroup distance for the *COI* sequences. The intraspecific pairwise distances were generally less than 2%, with an exception for *S. watanabei* (3.1%) and *Scolia superciliaris* (0–4%), both of which consist of two subspecies among the studied specimens. The interspecific pairwise distance ranged from 5.3% to 20.8%.

**Figure 1:**
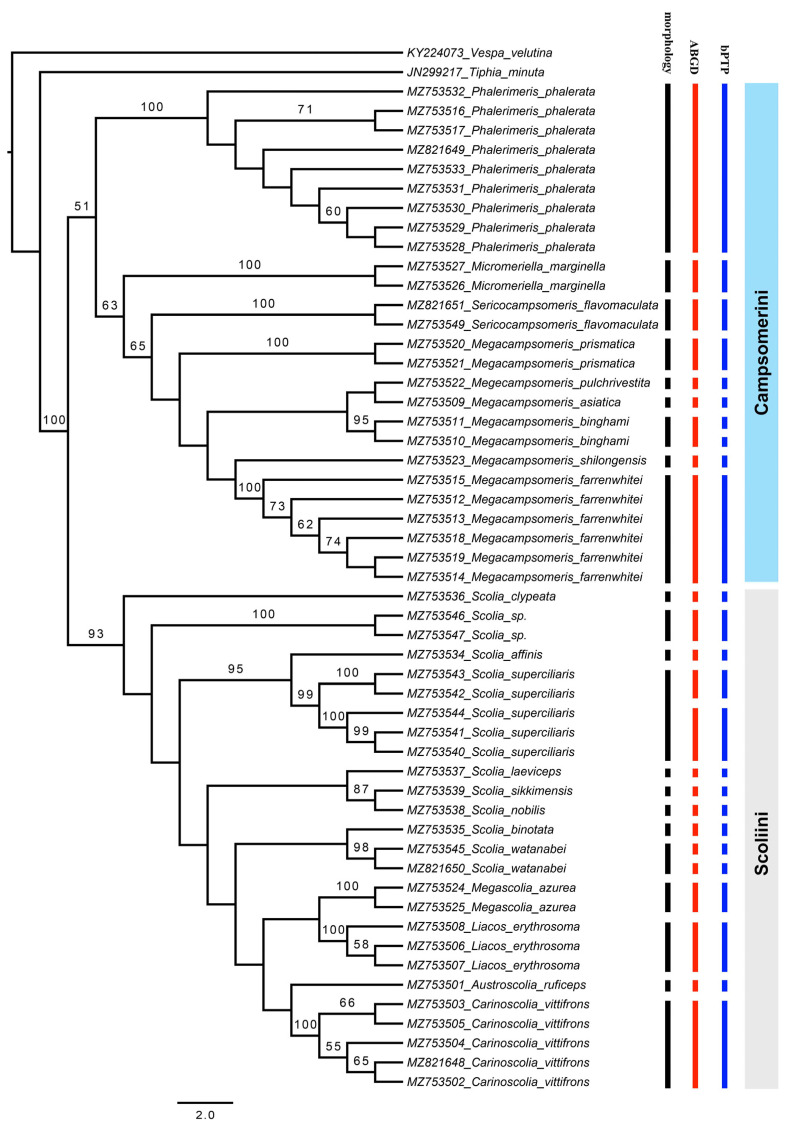
Maximum likelihood tree based on *COI* and results of species delimitation of three methods, only values > 50 for bootstrap are labeled.

The ABGD analyses returned a total of 24 groups at a priori genetic distance thresholds of 0.002–0.036. The two subspecies of *S. watanabei* and *S. superciliaris* were assigned as putative species. After removing the outgroups, the bPTP analysis based on the ML tree delimited 25 putative species. Besides the two subspecies of *S. watanabei* and *S. superciliaris*, *Megacampsomeris binghami* was also recovered and consists of two putative species. The delimitations of all other species are congruent with the morphological identification results in both ABGD and bPTP methods (Figure 1).

**Figure 2:**
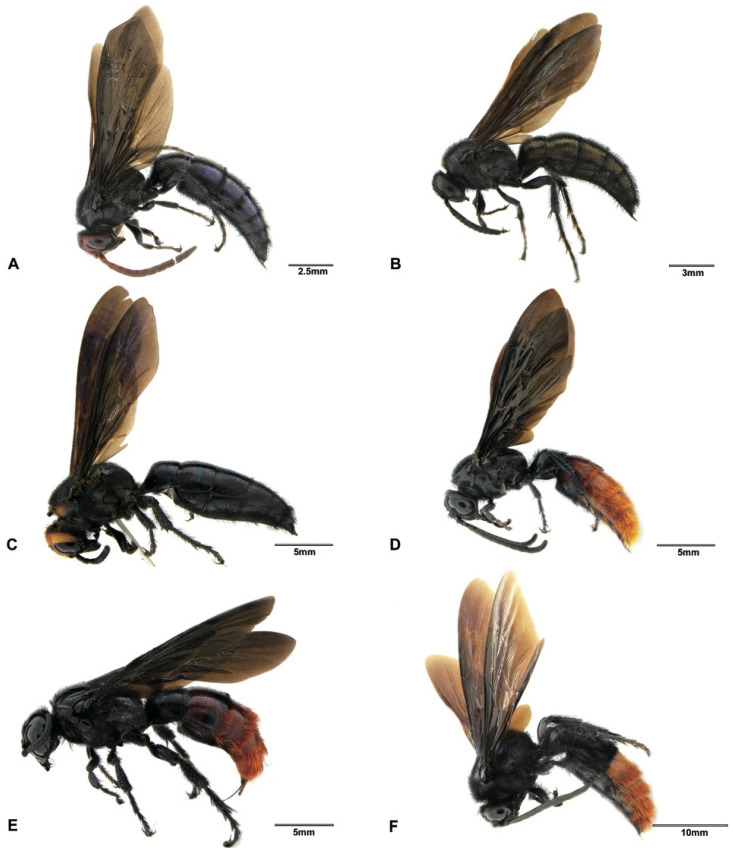
Habitus, lateral view. (**A**) *Austroscolia ruficeps* (Smith, 1855), male (SCAU3043675); (**B**) *Carinoscolia vittifrons* (Sichel, 1864), male (SCAU3043670); (**C**) *Carinoscolia vittifrons* (Sichel, 1864), female (SCAU3043672); (**D**) *Liacos erythrosoma* (Burmeister, 1853), male (SCAU3043662); (**E**) *Liacos erythrosoma* (Burmeister, 1853), female (SCAU3043661); (**F**) *Megascolia azurea* (Christ, 1791), male (SCAU3043666).

**Figure 3:**
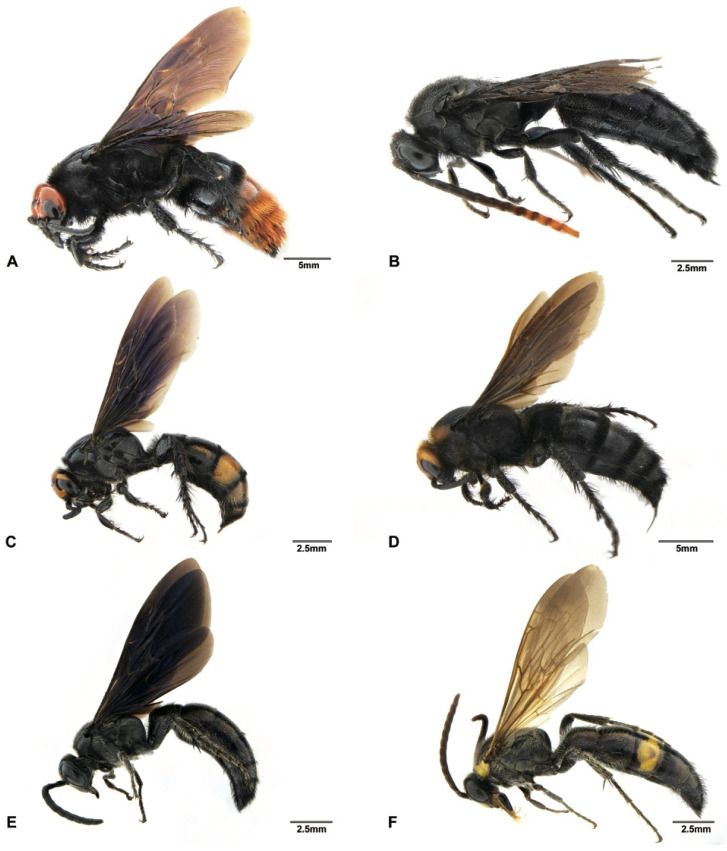
Habitus, lateral view. (**A**) *Megascolia azurea* (Christ, 1791), female (SCAU3043665); (**B**) *Scolia affinis* Guérin-Méneville, 1845, male (SCAU3043683); (**C**) *Scolia binotata* Betrem, 1928, female (SCAU3043684); (**D**) *Scolia clypeata* Sickman, 1894, female (SCAU3043682); (**E**) *Scolia laeviceps* Smith, 1855, male (SCAU3043680); (**F**) *Scolia* (*Discolia*) *nobilis* Saussure, 1858, male (SCAU3043681).

**Figure 4:**
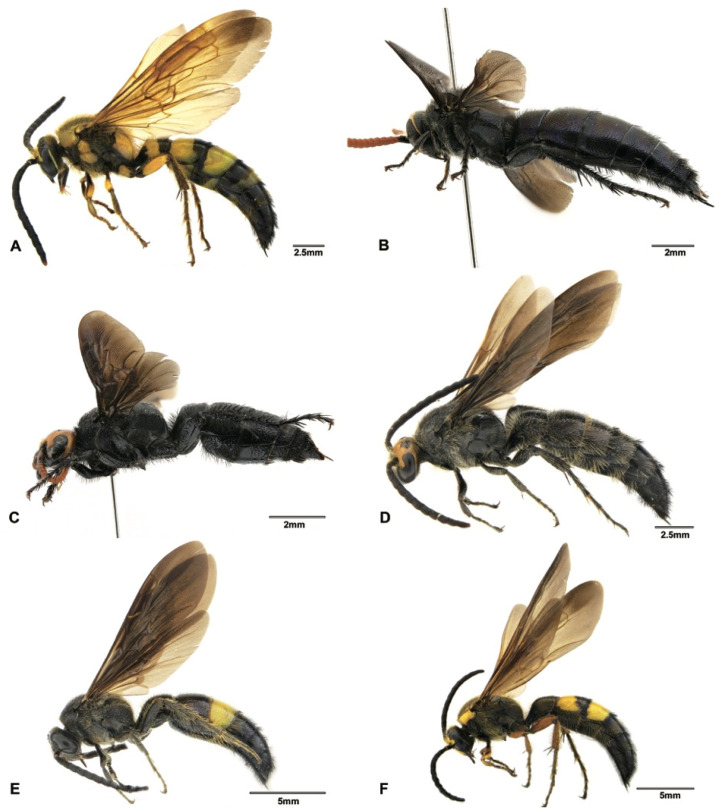
Habitus, lateral view. (**A**) *Scolia sikkimensis* Bingham, 1896, male (SCAU3048008); (**B**) *Scolia superciliaris sauteri* Betrem, 1928, male (SCAU3043677); (**C**) *Scolia superciliaris sauteri* Betrem, 1928, female (SCAU3043676); (**D**) *Scolia superciliaris superciliaris* Saussure, 1864, male (SCAU3048010); (**E**) *Scolia watanabei* Smith, 1855, male (SCAU3043679); (**F**) *Scolia watanabei* Smith, 1855, male (En-418585).

**Figure 5:**
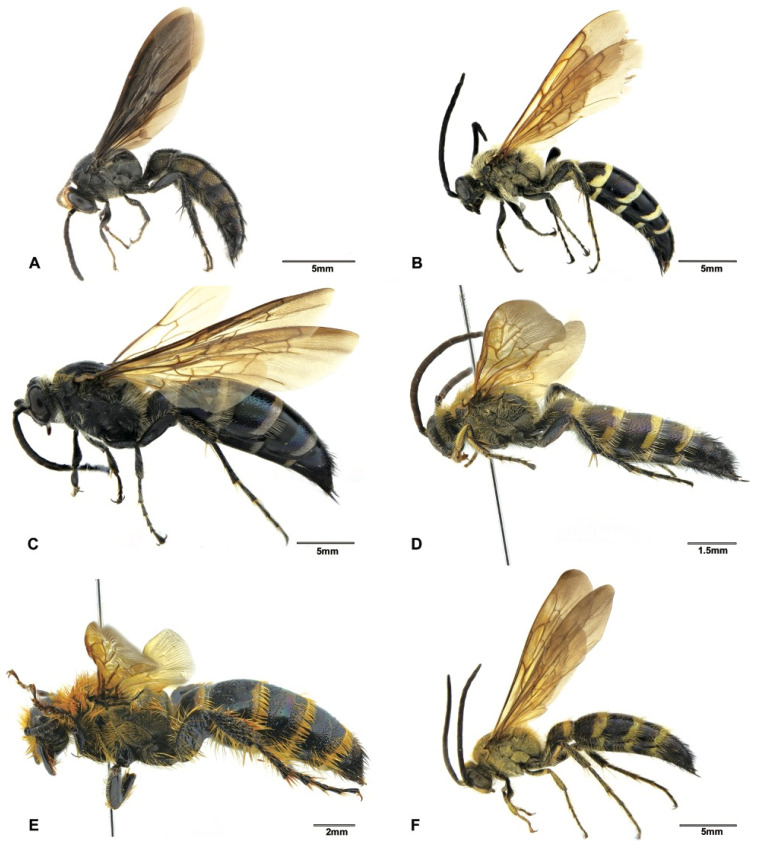
Habitus, lateral view. (**A**) *Scolia* sp., male (SCAU3043674); (**B**) *Megacampsomeris asiatica* (Saussure, 1859), male (SCAU3043656); (**C**) *Megacampsomeris binghami* (Betrem, 1928), male (SCAU3043660); (**D**) *Megacampsomeris farrenwhitei* Betrem, 1928, male (SCAU3043654); (**E**) *Megacampsomeris farrenwhitei* Betrem, 1928, female (SCAU3043653); (**F**) *Megacampsomeris prismatica* (Smith, 1855), male (SCAU3048015).

**Figure 6:**
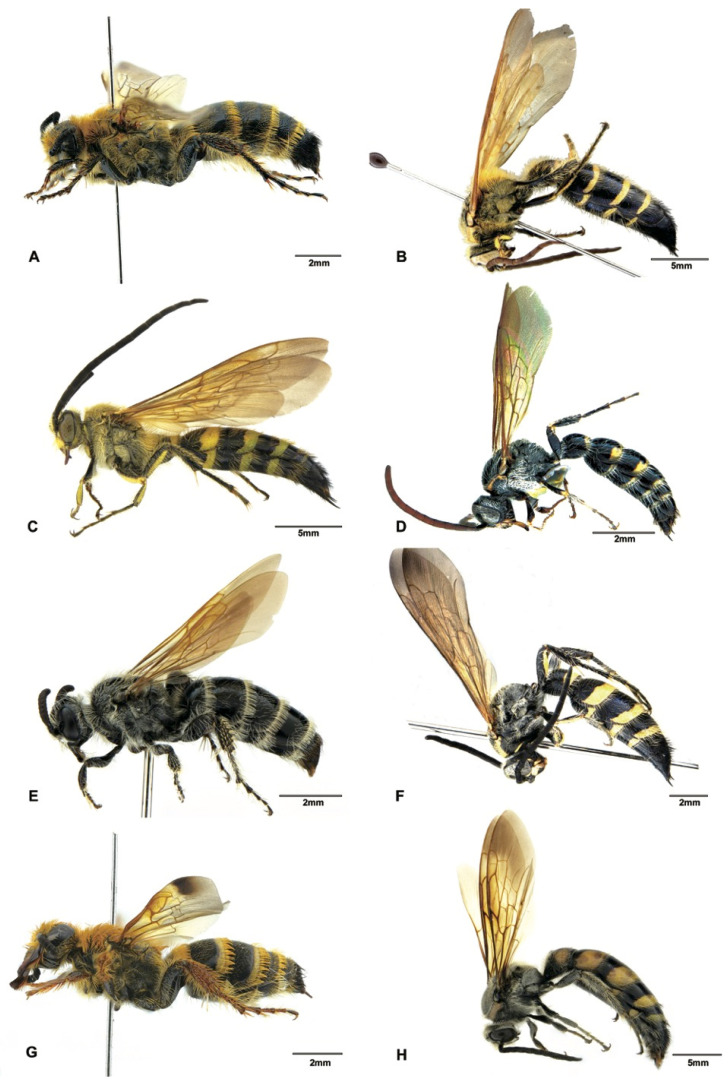
Habitus. (**A**) *Megacampsomeris prismatica* (Smith, 1855), female (SCAU3043653); (**B**) *Megacampsomeris pulchrivestita* (Cameron, 1902), male (NZ4760); (**C**) *Megacampsomeris shillongensis* Betrem, 1928, male (SCAU3043655); (**D**) *Micromeriella marginella* (Klug, 1810), male (SCAU3043668); (**E**) *Micromeriella marginella* (Klug, 1810), female (SCAU3043667); (**F**) *Phalerimeris phalerata* (Saussure,1858), male (SCAU3043685); (**G**) *Phalerimeris phalerata* (Saussure,1858), female (SCAU3043686); (**H**) *Sericocampsomeris flavomacula* Gupta & Jonathan, 1989, male (SCAU3043664).

Although the main goal of this study was not to address the phylogenetic relationships within the subfamily Scoliinae, our molecular data seem to support the tribal relationships proposed by Day et al. [30] (Figure 1) Tribe Scoliini, including genera *Austroscolia* (Figure 2A), *Carinoscolia* (Figure 2B,C), *Liacos* (Figure 2D,E), *Megascolia* (Figure 2F and Figure 3A), and *Scolia* (Figure 3B–F, Figure 4A–F and Figure 5A), form a highly (bootstrap = 93) supported clade. While Campsomerini, including genera *Megacampsomeris* (Figure 5B–F and Figure 6A–C), *Micromeriella* (Figure 6D,E), *Phalerimeris* (Figure 6F,G), and *Sericocampsomeris* (Figure 6H), form a relatively low (bootstrap = 51) supported clade.

### 3.1. Tribe Scoliini

Of the five genera within Scoliini, their interrelationships are still not clear because the branch supports among the genera are generally low (boostap < 50). Studies have found that *COI* has a low signal for phylogenetic analysis above the genus level [31]. Morphologically, the interrelationships within Scoliini are also unclear, for example, genera such as *Carinoscolia*, *Megascolia* and *Liacos* share many similarities and were treated as subgenera of *Scolia* in the past [18]. However, each species (some are represented by multiple specimens) recovered on the tree are clearly separated from all neighboring species and are identical to the number of species identified based on morphological characters, suggesting that the species in question can be identified unambiguously by DNA barcoding.

Eight determined and one undetermined species of *Scolia* were obtained in this study. *Scolia* (*Discolia*) *sikkimensis* Bingham (Figure 6C), which has been reported from Bhutan, India and Nepal [32], is newly recorded for China. The male of this species can be recognized by the presence of a distinct antero-median tubercle on the first abdominal tergite [32], as well as by the large degree of yellow maculation including the obvious markings on mesoscutum, mesopleurum, scutellum, propodeum and femura.

**Figure 7:**
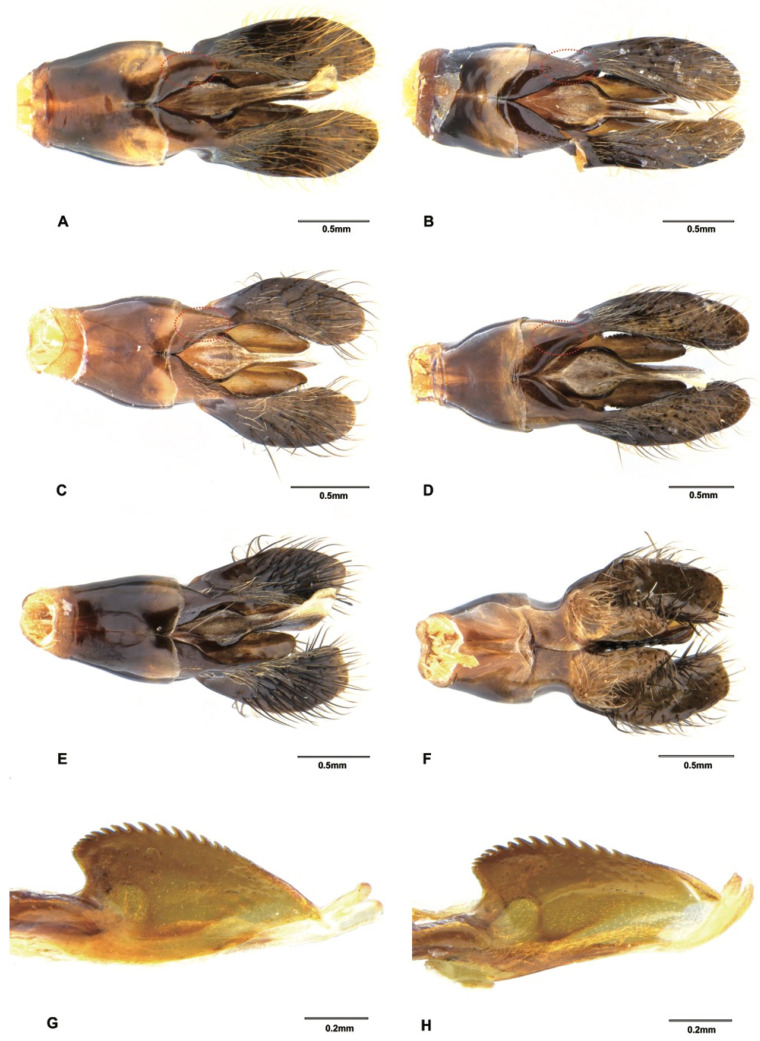
Male genitalia. (**A**) *Scolia watanabei* Smith, 1855 (En-418585), dorsal view; (**B**) *Scolia watanabei* Smith, 1855 (SCAU3043679), dorsal view; (**C**) *Scolia superciliaris sauteri* Betrem, 1928, dorsal view (SCAU3043677); (**D**) *Scolia superciliaris superciliaris* Saussure, 1864, dorsal view (SCAU3048010); (**E**) *Scolia* sp., (SCAU3043674) dorsal view; (**F**) *Scolia* sp., (SCAU3043674) ventral view; (**G**) *Megacampsomeris shillongensis* Betrem, 1928 (SCAU3043655), penis valve, lateral view; (**H**) *Megacampsomeris prismatica* (Smith, 1855) (SCAU3048015), penis valve, lateral view.

*Scolia (Discolia) watanabei* (Matsumura, 1912) is a polytypic species that is widely distributed in China, India, Japan and Myanmar [18]. The male of this species is easily confused with the males of *S. oculata* (Matsumura) and *S. hirta* (Schrank). This is also revealed in the blast results, that *S. watanabei* received close matches with *S. oculata* (over 97%) and *S. hirta* (over 96%) when blasted in the BOLD and GenBank databases. However, there are some characters that can be of diagnostic value: head with variable yellow patterns (all black in *S. oculata* and *S. hirta*), propodeum with larger and denser punctures and vestitures on mesosoma are golden (whitish or blackish in *S. oculata* and *S. hirta*). Three subspecies of *S. watanabei*, *viz*. *watanabei*, *pekingensis*, and *kempi*, have been reported and the main distinctive features between these subspecies are the variations in body markings, which were also recognized in a pending subspecies from Japan [33]. Two subspecies of *S. watanabei* were assigned as putative species from our ABGD and bPTP analyses. The male specimen from Hainan (SCAU3043679, Figure 4E) can be assigned to *pekingensis* for its color patterns (but subvertex without yellow transversal linear macula). The male specimen from Yunnan (En-418585, Figure 4F) differs from *pekingensis*, mainly in color patterns: Yellow maculae on clypeus, frons, scapula, fore legs and terga are larger; mid and hind femora mostly reddish. We also observed some differences between the male genitalia between this specimens and *pekingensis*: the squama of this specimen is polished (Figure 7A), but in *pekingensis*, the dorsal side of squama has some short and sparse hairs (Figure 7B). Considering the morphological distinctiveness and a relatively high genetic distance (3.1%), the specimens from Yunnan may represent a new subspecies or species. However, we need more morphological and molecular data from specimens, especially the females of the three known subspecies and the pending subspecies to confirm its identity.

*Solia superciliaris* is also a polytypic species that is widely distributed in China, India, Japan, Myanmar, Nepal, Thailand and Vietnam [18]. It is one of the most abundant species collected from southern China during this campaign. Betrem [17] recorded three subspecies, *viz*. *superciliaris*, *sauteri* and *staudingeri*. These subspecies are distinguished from each other mainly by the extent of the reddish and black color of the antennae, especially in males [31]. In this study, we found two typical subspecies (Figure 4B–D), namely *sauteri* and *superciliaris*, which can be easily distinguished by their red (SCAU3043676, SCAU3043677 and SCAU3048013) and black (SCAU3043678, SCAU3048010) antennae, respectively. In addition, vestiture is predominantly black on mesosoma and metasoma in subspecies *sauteri*, while it is white in subspecies *superciliaris*. The male genitalia also show similar differences, as found in the two subspecies of *S*. *watanabei* mentioned above: the squama of *sauteri* has short hairs (Figure 7C) but are polished in *superciliaris* (Figure 7D). These two subspecies were also supported by the ABGD and bPTP analyses (Figure 1).

Two undetermined male specimens (SCAU3043674 (Figure 5A), SCAU3048014) from Hainan are extremely similar to the males of *S. superciliaris superciliaris.* Only some subtle differences can be found: an ocelli part reddish yellow (black in *S. superciliaris superciliaris*); vestiture predominantly black on mesoscutum and metasoma (whitish in *S. superciliaris superciliaris*); punctures on propodeum and scutellum are coarser and denser, interspaces between punctures are sometimes smaller than the puncture diameter (finer and sparser, interspaces between punctures always distinctly bigger than the puncture diameter in *S. superciliaris superciliaris*); and metasoma iridescent with a coppery sheen (less shiny in *S. superciliaris superciliaris*). Male genitalia, however, are distinctly different from the two subspecies of *S. superciliaris* mentioned above: hairs on paramere are thicker and black and paramere truncated apically (Figure 7E,F), although the sparse and short hairs present on squama are similar to *S. superciliaris superciliaris sauteri*. Plus, the *COI* sequences provide strong evidence that these are different species, with a 7.7–8.6% genetic distance between the two species. This genetic distance matches the level of genetic divergence of mtDNA between animal species (8–17%) [34]. This indicates that cryptic species may exist in *Scolia* and male genitalia and DNA barcoding are useful in recognizing these species.

### 3.2. Tribe Campsomerini

Of the four genera within Campsomerini, the generic status seems to be supported by the phylogenetic analysis of the *COI* sequences, as the branches representing each genus are clearly separated from each other (Figure 1). Genera *Phalerimeris* Betrem, *Micromeriella* Betrem, and *Sericocampsomeris* Betrem previously were all elevated from subgenera of *Campsomeris* [18]. In this study, these genera are all represented by one species but with multiple specimens collected from various localities and form their own clusters that are clearly separated from each other on the ML tree.

*Sericocampsomeris flavomacula* Gupta and Jonathan, 1989 (Figure 6H) was previously reported from India and Nepal, and here we record this species from China for the first time. This species can be distinguished from the two *Sericocamposomeris* species previously recorded in China by its large extent of reddish-yellow maculation on the first to fifth terga and its predominantly white vestiture.

Genus *Megacampsomeris* Betrem, however, is diverse and six species are included in this study. Males of *Megacampsomeris* are extremely morphologically similar to each other. However, the results from the ABGD and bPTP analyses are generally consistent with the results based on morphological identification.

Three species of *Megacampsomeris*, i.e., *Megacampsomeris asiatica* (Saussure, 1858), *Megacampsomeris pulchrivestita* (Cameron, 1902) and *Megacampsomeris shillongensis* (Betrem, 1928), are newly recorded in China. These three species are all widely distributed in the Oriental region [16,32]. The male of *M. asiatica* (Figure 5B) can be differentiated from other species by having the integument of the head and mesosoma entirely black and the vestiture distinctly white on mesosoma; the male of *M. pulchrivestita* (Figure 6B) can be recognized by its clypeus yellow, vestiture reddish yellow on mesosoma, fringes of terga white and short, narrow bands on terga, and completely black cutellum; while the male of *M. shillongensis* (Figure 6C) can be separated from other species by their yellow clypeus, largely yellow femora on the outer surface, apical bands on terga broad, golden vestiture, other than the black on the fifth to last abdominal segments, and the penis valve of male genitalia are less elongate apicad (Figure 7G) when compared with its congener *M. prismatica* (Figure 7H).

**Figure 8:**
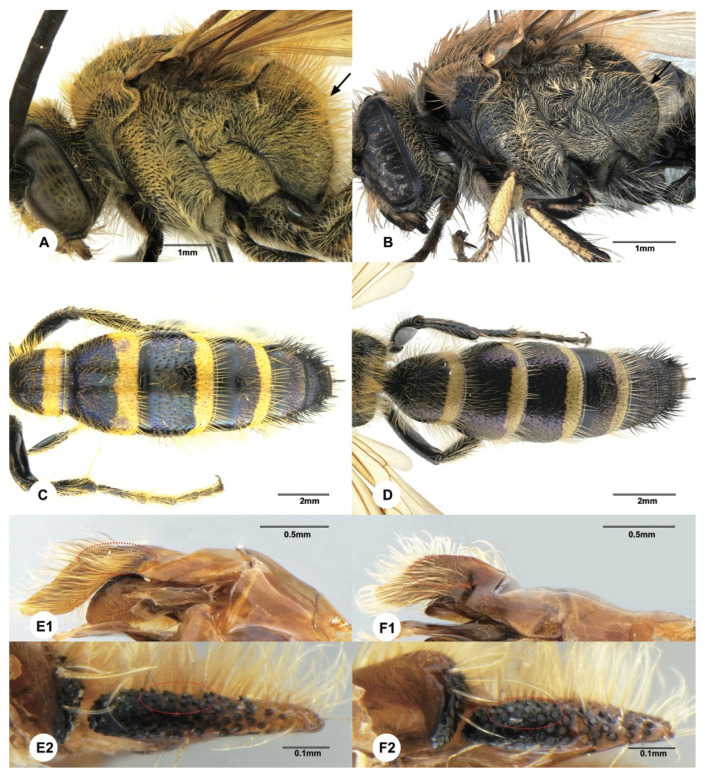
(**A**,**C**,**E1**,**E2**): Male of *Megacampsomeris prismatica* (Smith, 1855) (SCAU3048015); (**B**,**D**,**F1**,**F2**): *Megacampsomeris farrenwhitei* Betrem, 1928 (SCAU3043654). (**A**,**B**). mesopleurum, lateral view; (**C**,**D**). metasoma, dorsal view; (**E1**,**F1**). paramere of male genita; (**E2**,**F2**). volsella of male genita.

In some cases, DNA barcoding could enhance morphological species delimitation by assisting in discovering useful diagnostic morphological characters. Morphologically, the males of *Megacampsomeris prismatica* (Smith, 1855) and *Megacampsomeris farrenwhitei* (Betrem, 1928) are easily confused with each other. These two species are sympatric and commonly occur in southern China, but the males have never been thoroughly described. According to the literature [16,23,32], the most distinctive character for males of these two species could be the yellow marking on the hind femur (*M. prismatica* is all black while *M. farrenwhitei* is with yellow stripe beneath the hind femur). However, the male specimens of *M. prismatica* from Japan were found to be variable in maculation, sometimes even with a yellow stripe beneath the hind femur [35]. Females (Figure 5E and Figure 6A) of these species are easily distinguished by the punctuation on scutellum, coloration of wings and median grove of frons [23]. In this study, males of these two species were assigned to the respective species based on their identities to the female *COI* sequences. After such assignment, the intraspecific genetic distances of the two species are not more than 0.3%, while the interspecific genetic distance between these two species ranged between 13% to 13.5%. Further morphological examination indicates that the yellow marking on the hind femur of *M*. *farrenwhitei* is variable from presentation beneath the hind femur (e.g., SCAU3043654, SCAU3043659) to being entirely absent (e.g., SCAU3043658). Therefore, the yellow marking on the hind femur is not a valid characteristic by which to differentiate the males of these two species. However, based on the molecular species delimitation, we found that some characters (Figure 8A–F) might be of diagnostic value: vestiture on mesosoma is denser and more golden in *M. prismatica*, while it is sparser and more reddish in *M. farrenwhitei*; the mid subhorizontal portion of propodeum, in the lateral view, slopes rather steeply, making a sharper angle with the vertical portion in *M. prismatica*, while it is more gradual, making an obtuse angle in *M. farrenwhitei*; yellow bands on terga are wider (nearly 1/3 of mid length) in *M. prismatica* while they are narrower (about 1/4 of mid length) in *M. farrenwhitei*; and pubescence thicker on paramere and volsella is more acutely dentated in *M. prismatica*, while pubescence is sparser and volsella is more obtusely dentated in *M. farrenwhitei.* This example indicates that DNA barcoding is useful to recognize these problematic species in Scoliidae.

The two specimens of *Megacampsomeris binghami* (Betrem, 1928) were recovered as two putative species by the bPTP analysis. However, the pairwise distance between these two specimens was 1.9% and few morphological differences were observed. This relatively high genetic variation is probably due to their distant geographic distribution, as one specimen (SCAU3048011) was collected in Guangdong and the other (SCAU3043660, Figure 5C) in the mountainous area of Yunnan. Besides, studies have shown that the bPTP methods tend to oversplit species [36].

## 4. Conclusions

The scoliid wasps from southern China are polymorphic and diverse. Our analyses based on both molecular data and morphology recognized 22 species belonging to 9 genera of two tribes, including 5 species newly recorded for China: *S.* (*Discolia*) *sikkimensis*, *S. flavomaculata*, *M. asiatica*, *M. pulchrivestita* and *M. shillongensis*. The phylogenetic tree based on the *COI* sequences shows perfect consistence with the tribal system of Day et al. [30] and the general classification of the Chinese Scoliidae proposed by Liu et al. [23], suggesting that the *COI* sequences provide a relatively accurate picture of evolutionary history. Furthermore, DNA barcoding is not only useful in discovering cryptic species, but also in delimitating morphologically similar species. Our study indicates that the integrative approach, combing both molecular and morphological evidence, is a potent tool to tackle the taxonomic challenges in the family Scoliidae, of which sexual dimorphism and cryptic species are common.

## Figures and Tables

**Table 1:** Sequenced taxa and GenBank accession numbers.

Code	Species	Sex	GenBank Accession No.
SCAU3043675	*Austroscolia ruficeps* (Smith)	male	MZ753501
SCAU3043670	*Carinoscolia vittifrons* (Sichel)	male	MZ753502
SCAU3043671	*Carinoscolia vittifrons* (Sichel)	female	MZ753503
SCAU3043672	*Carinoscolia vittifrons* (Sichel)	female	MZ753504
SCAU3043673	*Carinoscolia vittifrons* (Sichel)	male	MZ753505
En-418584	*Carinoscolia vittifrons* (Sichel)	female	MZ821648
SCAU3043661	*Liacos erythrosoma* (Burmeister)	female	MZ753506
SCAU3043662	*Liacos erythrosoma* (Burmeister)	male	MZ753507
SCAU3048016	*Liacos erythrosoma* (Burmeister)	male	MZ753508
SCAU3043656	*Megacampsomeris asiatica* (Saussure)	male	MZ753509
SCAU3043660	*Megacampsomeris binghami* (Betrem)	male	MZ753510
SCAU3048011	*Megacampsomeris binghami* (Betrem)	male	MZ753511
SCAU3043653	*Megacampsomeris farrenwhitei* (Betrem)	female	MZ753512
SCAU3043654	*Megacampsomeris farrenwhitei* (Betrem)	male	MZ753513
SCAU3043658	*Megacampsomeris farrenwhitei* (Betrem)	male	MZ753514
SCAU3043659	*Megacampsomeris farrenwhitei* (Betrem)	male	MZ753515
SCAU3043686	*Megacampsomeris farrenwhitei* (Betrem)	female	MZ753517
SCAU3048009	*Megacampsomeris farrenwhitei* (Betrem)	male	MZ753518
SCAU3048012	*Megacampsomeris farrenwhitei* (Betrem)	male	MZ753519
SCAU3043657	*Megacampsomeris prismatica* (Smith)	female	MZ753520
SCAU3048015	*Megacampsomeris prismatica* (Smith)	male	MZ753521
NZ4760	*Megacampsomeris pulchrivestita* (Cameron)	male	MZ753522
SCAU3043655	*Megacampsomeris shillongensis* (Betrem)	male	MZ753523
SCAU3043665	*Megascolia* (*Regiscolia*) *azurea* (Christ)	female	MZ753524
SCAU3043666	*Megascolia* (*Regiscolia*) *azurea* (Christ)	male	MZ753525
SCAU3043667	*Micromeriella marginella* (Klug)	female	MZ753526
SCAU3043668	*Micromeriella marginella* (Klug)	male	MZ753527
SCAU3043685	*Phalerimeris phalerata* (Saussure)	male	MZ753516
NZ4762	*Phalerimeris phalerata* (Saussure)	female	MZ753528
NZ4792	*Phalerimeris phalerata* (Saussure)	male	MZ753529
NZ4832	*Phalerimeris phalerata* (Saussure)	male	MZ753530
NZ4835	*Phalerimeris phalerata* (Saussure)	female	MZ753531
SCAU3043669	*Phalerimeris phalerata* (Saussure)	male	MZ753532
SCAU3048017	*Phalerimeris phalerata* (Saussure)	male	MZ753533
En-418587	*Phalerimeris phalerata* (Saussure)	female	MZ821649
SCAU3043683	*Scolia* (*Discolia*) *affinis* Guérin-Méneville	male	MZ753534
SCAU3043684	*Scolia* (*Discolia*) *binotata* Fabricius	female	MZ753535
SCAU3043682	*Scolia* (*Discolia*) *clypeata* Sickman	female	MZ753536
SCAU3043680	*Scolia* (*Discolia*) *laeviceps* Smith	male	MZ753537
SCAU3043681	*Scolia* (*Discolia*) *nobilis* Saussure	male	MZ753538
SCAU3048008	*Scolia* (*Discolia*) *sikkimensis* Bingham	male	MZ753539
SCAU3043676	*Scolia* (*Discolia*) *superciliaris* Saussure	female	MZ753540
SCAU3043677	*Scolia* (*Discolia*) *superciliaris* Saussure	male	MZ753541
SCAU3043678	*Scolia* (*Discolia*) *superciliaris* Saussure	male	MZ753542
SCAU3048010	*Scolia* (*Discolia*) *superciliaris* Saussure	male	MZ753543
SCAU3048013	*Scolia* (*Discolia*) *superciliaris* Saussure	male	MZ753544
SCAU3043679	*Scolia* (*Discolia*) *watanabei* (Matsumura)	male	MZ753545
En-418585	*Scolia* (*Discolia*) *watanabei* (Matsumura)	male	MZ821650
SCAU3043674	*Scolia* sp.	male	MZ753546
SCAU3048014	*Scolia* sp.	male	MZ753547
SCAU3043664	*Sericocampsomeris flavomacula* Gupta and Jonathan	male	MZ753549
En-418591	*Sericocampsomeris flavomacula* Gupta and Jonathan	male	MZ821651

## Data Availability

The data of the research were deposited in the Museum of Biology at Sun Yat-sen University (SYSBM), Guangzhou, China.

## Supplementary Materials

The following are available online at https://www.mdpi.com/article/10.3390/insects12100892/s1, Table S1: Details of sequenced specimens, Table S2: Genetic distance of *COI* within species under K2P model, Table S3: Interspecific pairwise distance of Scollidae based on *COI* sequences (%).
