# Supplementary material for: Tackling the Taxonomic Challenges in the Family Scoliidae (Insecta, Hymenoptera) Using an Integrative Approach: A Case Study from Southern China
Source: Insects. 2021 Oct 1;12(10):892. doi: 10.3390/insects12100892 (PMC8539399; doi:10.3390/insects12100892)
Supplement: Supplementary file 1 [file insects-12-00892-s001.zip › Table S3.pdf]

Table S3 Interspecific pairwise distance of Scollidae based on COI sequences (%)

Species	1	2	3	4	5	6	7	8	9	10	11	12	13	14	15	16	17	18	19	20	21	22
1 *Austroscolia ruficeps*		18.7–18.8	9.8–10	19.2	19–19.2	20.3–20.7	19.3–19.4	18.1	17.1	7.2–7.4	17.4	15.3–16.4	8.4	8.8	9.4	9.9	7.6	8.8	9.5–9.9	7.9–9	9.1–9.3	19.9–20
2 *Carinoscolia vittifrons*			10.2–11.2	19.1–19.5	18.7–19.2	20.4–21.2	18.9–19.8	19.8–20.4	17.6–18.1	9.3–10	18.9–19.5	16.4–17.8	20.3–21.1	10–10.6	20.2–20.9	20.6–20.9	20.2–21.1	10.9–11.4	10.9–12	10.5–11.7	9.7–10.6	20.4–21.2
3 *Liacos erythrosoma*				20.3–20.4	19.2–19.6	19.6–20.3	18.7–19.1	18.4	17.4–17.5	8.1–8.5	17.8–18.1	15.1–16.5	10.7–11	9.7–10	10.4–10.5	10.1–10.4	10–10.2	10.3–10.6	10.6–11.2	9.1–10.6	10–10.5	11.1–11.5
4 *Megacampsomeris asiatica*					9.1–9.5	15.3–15.6	10.6–10.8	10.7	9.4	9.3–9.4	17.4	14.9–15.4	9.1	19.6	19.4	18.1	18.5	20.1	19.6–20.7	19.2–19.4	19.3–19.4	16.7–16.8
5 *Megacampsomeris binghami*						14.1–14.3	8.9–9.1	8.3–8.8	9.1–9.7	17.9–18	16.8–17.1	13.4–13.8	18.6–18.7	18.5–18.7	19.5	18.9	18.6–18.7	19.8–19.9	18.6–19	18.4–19.2	18.2–18.6	15.5–16.2
6 *Megacampsomeris farrenwhitei*							13–13.5	14.7–15	22.1–22.7	18–18.5	17.6–17.9	15.5–16.7	18.8–19.1	19.1–19.3	19.3–19.8	20.1–20.4	19.9–20.4	20.3–20.6	19.7–20.2	20–20.6	17.9–19.4	19.2–19.7
7 *Megacampsomeris prismatica*								9	8.1–8.4	16.5–16.7	16.7–17.1	13.2–14.4	18–18.1	18–18.1	18–18.1	17.6	17.4–17.5	17.8–18	16.7–18	17.1–18.1	16.7–16.9	16.2–16.4
8 *Megacampsomeris pulchrivestita*									9	17.1–17.4	15	14.2–15	18.9	18.6	18.4	17.7	17.4	18.9	17.8–18.6	18.1–18.7	18–18.1	15.5–15.6
9 *Megacampsomeris shillongensis*										15.3–15.5	15.2–15.3	13.6–14.2	16.4	17.4	17.9	17.7	17.3	17.9	15.9–16.9	17.2–17.9	16.8–16.9	15.8–15.9
10 *Megascolia* (*Regiscolia*) *azurea*											17.1–17.2	13.9–15	18.4	8.5–8.6	9.7–9.8	8.7	8.1	9–9.1	8.2–8.5	7–8.4	8.1–8.3	19.8–20
11 *Micromeriella marginella*												15.5–16.4	17.9	17.1	17.7–17.8	18–18.1	17.8	18.5	17.9–18.8	17.6–18.4	16.7–17.1	18.8–18.9
12 *Phalerimeris phalerata*													14.2–15.3	15.2–16.4	15.1–16.2	15.5–16.4	14.8–15.7	16–17	14.5–16	15–16.7	13.7–14.8	16.5–17.4
13 *Scolia* (*Discolia*) *affinis*														9.1	8.5	6.9	7.9	8.5	5.9–6.3	7.3–7.5	5.7–5.9	19.5–19.6
14 *Scolia* (*Discolia*) *binotata*															8.2	8.9	9.2	10.1	10–10.9	7.4–8.8	7.8	19.6–19.7
15 *Scolia* (*Discolia*) *clypeata*																8.5	8.3	9.7	9.5–10	9.1–9.8	6.9–7.4	8.6–8.7
16 *Scolia* (*Discolia*) *laeviceps*																	6	7.6	8.5–8.7	7.3–7.8	6.2–6.3	19.9–20
17 *Scolia* (*Discolia*) *nobilis*																		5.3	8.3–9.3	6.5–7.6	6.6–6.7	19–19.1
18 *Scolia* (*Discolia*) *sikkimensis*																			8.8–9.1	7.6–8.5	7.2–7.7	20.5–20.6
19 *Scolia* (*Discolia*) *superciliaris*																				7.8–9.1	7.7–8.6	20.2–20.8
20 *Scolia* (*Discolia*) *watanabei*																					6.2–7.6	8.9–9.1
21 *Scolia* sp.																						19.7–19.9
22 *Sericocampsomeris flavomaculata*																						
