# Supplementary material for: Tackling the Taxonomic Challenges in the Family Scoliidae (Insecta, Hymenoptera) Using an Integrative Approach: A Case Study from Southern China
Source: Insects. 2021 Oct 1;12(10):892. doi: 10.3390/insects12100892 (PMC8539399; doi:10.3390/insects12100892)
Supplement: Supplementary file 1 [file insects-12-00892-s001.zip › Tables S1 & S2.pdf]

Supplement

Table S1 Details of sequenced specimens

Code	Species	Collecting data
SCAU3043675	*Austroscolia ruficeps*	Hainan, Mt. Diaoluoshan, 18°39'55.6"N109°58'16.04"E, 30.vi-31.vii.2020, Malaise trap, Longlong Chen
SCAU3043670	*Carinoscolia vittifrons*	Guangdong, Mt. Dinghushan, 23°9'48.02"N112°32'52.03"E, 29.iv-31.v.2020, Malaise trap, Longlong Chen
SCAU3043671	*Carinoscolia vittifrons*	Guangdong, Mt. Dinghushan, 23°9'48.02"N112°32'52.03"E, 29.iv-31.v.2020, Malaise trap, Longlong Chen
SCAU3043672	*Carinoscolia vittifrons*	Guangdong, Mt. Dinghushan, 23°9'48.02"N112°32'52.03"E, 29.iv-31.v.2020, Malaise trap, Longlong Chen
SCAU3043673	*Carinoscolia vittifrons*	Guangdong, South China Botanical Garden, 23°11'25.82"N113°21'47.01"E, 1-30.vi.2020, Malaise trap, Jianwei Li
En-418584	*Carinoscolia vittifrons*	Guangdong, Mt. Dinghushan, 23°9'27.93"N112°32'45.46"E, 18.ix-23.x.202, Malaise trap
SCAU3043661	*Liacos erythrosoma*	Guangdong, Shenzhen, 22°31'59.58"N113°58'7.58"E, 29.iv-5.vi.2020, Malaise trap, Longlong Chen
SCAU3043662	*Liacos erythrosoma*	Guangdong, Shenzhen, 22°31'59.58"N113°58'7.58"E, 29.iv-5.vi.2020, Malaise trap, Longlong Chen
SCAU3048016	*Liacos erythrosoma*	Hainan, Nankai Town, areca-nut plantation, 19°4'44.68"N109°24'4.74"E, 31.i-29.ii.2020, Malaise trap, Longlong Chen
SCAU3043656	*Megacampsomeris asiatica*	Yunnan, Mt. Gaoligongshan, 26°0'52.55"N98°38'7.23"E, 2022m, 1-15.ix,2020, Malaise trap, Lang Yi
SCAU3043660	*Megacampsomeris binghami*	Yunnan, Mt. Gaoligongshan, 27°41'6.73"N98°16'25.64"E, 1181m,20.iv-15.v.2020, Lang Yi
SCAU3048011	*Megacampsomeris binghami*	Guangdong, Mt. Yunkaishan, 22°16'22.67"N111°11'38.7"E, 2-27.xi.2020, Malaise trap, Longlong Chen
SCAU3043653	*Megacampsomeris farrenwhitei*	Guangdong, South China Botanical Garden, 23°10'56.16"N113°21'28.94"E, 1-30.vi.2020, Malaise trap, Jianwei Li
SCAU3043654	*Megacampsomeris farrenwhitei*	Guangdong, South China Botanical Garden, 23°10'56.16"N113°21'28.94"E, 1-30.vi.2020, Malaise trap, Jianwei Li
SCAU3043658	*Megacampsomeris farrenwhitei*	Guangdong, Mt. Gutianshan, 23°6'51.42"N114°47'36.35"E, 30.x-30.xi.2019, Malaise trap, Longlong Chen
SCAU3043659	*Megacampsomeris farrenwhitei*	Guangdong, South China Botanical Garden, 23°10'49.87"N113°21'21.33"E, 1.viii-1.ix.2020, Malaise trap, Wei Lin
SCAU3043686	*Megacampsomeris farrenwhitei*	Guangdong, South China Botanical Garden, 23°10'56.83"N113°21'39.38"E, 2.xi-7.xii.2020, Malaise trap, Jianwei Li
SCAU3048009	*Megacampsomeris farrenwhitei*	Hainan, Mt. Bawangling, 19°7'16.59"N109°5'12.24"E, 30.vii-30.viii.2020, Malaise trap, Longlong Chen
SCAU3048012	*Megacampsomeris farrenwhitei*	Hainan, Mt. Limushan, 19°10'22.14"N109°43'10.34"E, 31.vii-1.ix.2020, Malaise trap, Longlong Chen
SCAU3043657	*Megacampsomeris prismatica*	Guangdong, Mt. Gutianshan, 23°6'51.42"N114°47'36.35"E, 30.x-30.xi.2019, Malaise trap, Longlong Chen
SCAU3048015	*Megacampsomeris prismatica*	Hainan, Mt. Jianfengling, 18°41'35.57"N108°46'59.25"E, 30.xii.2019-30.i.2020, Malaise trap, Chunyang Xu
NZ4760	*Megacampsomeris pulchrivestita*	Guangdong, Mt. Gutianshan, 23°6'51.42"N114°47'36.35"E, 22.ix.2019, sweep net, Longlong Chen
SCAU3043655	*Megacampsomeris shillongensis*	Yunnan, Mt. Gaoligongshan, 25°25'59.6"N98°41'32.4"E, 1739m, 16-30.xi.2019, Malaise trap, Lang Yi
SCAU3043665	*Megascolia* (*Regiscolia*) *azurea*	Hainan, Nankai Town, edge of forest, 19°4'20.77"N109°22'28.2"E, 31.i-29.ii.2020, Malaise trap, Longlong Chen
SCAU3043666	*Megascolia* (*Regiscolia*) *azurea*	Guangdong, Shenzhen, Xianhu Botanical Garden, 22°34'49.38"N114°10'22.01"E, 3-31.iii.2020, Malaise trap, Longlong Chen
SCAU3043667	*Micromeriella marginella*	Hainan, Wenchang, 19°32'56.61"N110°47'28.82" E, 10-20.ix,2019, Malaise trap, Chunyang Xu
SCAU3043668	*Micromeriella marginella*	Hainan, Wenchang, 19°32'56.61"N110°47'28.82" E, 10-20.ix,2019, Malaise trap, Chunyang Xu
SCAU3043685	*Phalerimeris phalerata*	Guangdong, South China Botanical Garden, 23°11'11.69"N113°21'49.52"E, 1.ix-9.x.2020, Malaise trap, Jianwei Li
NZ4762	*Phalerimeris phalerata*	Guangdong, Dongguan Botanical Garden, 22°57'45.54"N113°44'29.18"E, 24.ix.2019, sweep net, Longlong Chen

NZ4792	*Phalerimeris phalerata*	Guangdong, Dalingshan Forest Park, 22°51'30.23"N113°45'16.58"E, 30.vii.2020, sweep net, Longlong Chen
NZ4832	*Phalerimeris phalerata*	Guangdong, Mt. Gutianshan, 23°6'51.42"N114°47'36.35"E, 4.viii.2020, sweep net, Longlong Chen
NZ4835	*Phalerimeris phalerata*	Guangdong, Mt. Gutianshan, 23°6'51.42"N114°47'36.35"E, 4.viii.2020, sweep net, Longlong Chen
SCAU3043669	*Phalerimeris phalerata*	Guangdong, South China Botanical Garden, 23°11'11.69"N113°21'49.52"E, 30.vi-1.viii.2020, Malaise trap, Wei Lin
SCAU3048017	*Phalerimeris phalerata*	Hainan, Danzhou, 19°30'50.46"N109°30'25.32"E, 30.iv-30.v.2020, sweep net, Longlong Chen
En-418587	*Phalerimeris phalerata*	Guangdong, Mt. Dinghushan, 23°9'27.93"N112°32'45.46"E, 18.ix-23.x.202, Malaise trap
SCAU3043683	*Scolia* (*Discolia*) *affinis*	Guangdong, Mt. Xiangtoushan, 23°15'55.49"N114°26'50.46"E, 20-30.vii.2019, Malaise trap, Longlong Chen
SCAU3043684	*Scolia* (*Discolia*) *binotata*	Guangdong, Shenzhen, Xianhua Botanical Garden, 22°34'49.38"N114°10'22.01"E, 5-29.vi.2020, Malaise trap, Jianwei Li
SCAU3043682	*Scolia* (*Discolia*) *clypeata*	Guangdong, Shenzhen, 22°31'59.58"N113°58'7.58"E, 29.iv-5.vi.2020, Malaise trap, Longlong Chen
SCAU3043680	*Scolia* (*Discolia*) *laeviceps*	Hainan, Nankai Town, areca-nut plantation, 19°4'44.68"N109°24'4.74"E, 31.iii-30.iv.2020, Malaise trap, Longlong Chen
SCAU3043681	*Scolia* (*Discolia*) *nobilis*	Guangdong, Mt. Dinghushan, 23°9'48.02"N112°32'52.03"E, 29.iv-31.v.2020, Malaise trap, Longlong Chen
SCAU3048008	*Scolia* (*Discolia*) *sikkimensis*	Yunnan, Mt. Gaoligongshan, 25°18'22.99"N98°47'37.34"E, 1686m, 15—30.vi.2020, Malaise trap, Lang Yi
SCAU3043676	*Scolia* (*Discolia*) *superciliaris*	Guangdong, South China Botanical Garden, 23°10'50.6"N113°21'35.01"E, 30.vi-1.viii.2020, Malaise trap, Wei Lin
SCAU3043677	*Scolia* (*Discolia*) *superciliaris*	Guangdong, South China Botanical Garden, 23°10'56.83"N113°21'39.38"E, 30.vi-1.viii.2020, Malaise trap, Wei Lin
SCAU3043678	*Scolia* (*Discolia*) *superciliaris*	Guangdong, South China Botanical Garden, 23°10'56.83"N113°21'39.38"E, 30.vi-1.viii.2020, Malaise trap, Wei Lin
SCAU3048010	*Scolia* (*Discolia*) *superciliaris*	Guangdong, Dongguan, Mt. Yinpingshan, 22°54'26.09"N114°13'13.47"E, 30.iv-31.v.2020, Malaise trap, Longlong Chen
SCAU3048013	*Scolia* (*Discolia*) *superciliaris*	Hainan, Mt. Diaoluoshan, 18°40'2.4"N109°54'32.09"E, 31.vii-2.ix.2020, Malaise trap, Longlong Chen
SCAU3043679	*Scolia* (*Discolia*) *watanabei*	Hainan, Mt. Jianfengling, 18°42'14.26"N108°47'21.23"E, 89m, 30.x-30.xi.2019, Malaise trap, Chunyang Xu
En-418585	*Scolia* (*Discolia*) *watanabei*	Yunnan, Mt. Gaoligongshan, 25°18'22.99"N 98°47'37.34"E, 1686m, 15—30.vii.2020, Malaise trap, Lang Yi
SCAU3043674	*Scolia* sp.	Hainan, Mt. Diaoluoshan, 18°40'2.4"N109°54'32.09"E, 30.vi-31.vii.2020, Malaise trap, Longlong Chen
SCAU3048014	*Scolia* sp.	Hainan, Mt. Diaoluoshan, 18°40'2.4"N109°54'32.09"E, 31.vii-2.ix.2020, Malaise trap, Longlong Chen
SCAU3043664	*Sericocampsomeris flavomacula*	Hainan, Wenchang, 19°32'56.61"N110°47'28.82"E, 30.viii-10.ix.2019, Malaise trap, Chunyang Xu
En-418591	*Sericocampsomeris flavomacula*	Hainan, Wenchang, 19°32'56.61"N110°47'28.82"E, 30.viii-10.ix.2019, Malaise trap, Chunyang Xu

Table S2 Genetic distance of *COI* within species under K2P model

Species	Distance (%)
Austroscolia ruficeps	NA
Carinoscolia vittifrons	0-0.4
Liacos erythrosoma	0.7-1
Megacampsomeris asiatica	NA
Megacampsomeris binghami	1.9
Megacampsomeris farrenwhitei	0-0.3
Megacampsomeris prismatica	0.3
Megacampsomeris pulchrivestita	NA
Megacampsomeris shillongensis	NA
Megascolia (*Regiscolia*) *azurea*	0.1
Micromeriella marginella	0.1
Phalerimeris phalerata	0-0.6
Scolia (*Discolia*) *affinis*	NA
Scolia (*Discolia*) *binotata*	NA
Scolia (*Discolia*) *clypeata*	NA
Scolia (*Discolia*) *laeviceps*	NA
Scolia (*Discolia*) *nobilis*	NA
Scolia (*Discolia*) *sikkimensis*	NA
Scolia (*Discolia*) *superciliaris*	0-4
Scolia (*Discolia*) *watanabei*	3.1
Scolia sp.	0.1
Sericocampsomeris flavomaculata	0.1
